# City‐level water withdrawal in China: Accounting methodology and applications

**DOI:** 10.1111/jiec.12999

**Published:** 2020-03-09

**Authors:** Zongyong Zhang, Junguo Liu, Bofeng Cai, Yuli Shan, Heran Zheng, Xian Li, Xukun Li, Dabo Guan

**Affiliations:** 1 School of Environmental Science and Engineering, Southern University of Science and Technology, Shenzhen, 518055 China; 2 Water Security Research Centre, School of International Development, University of East Anglia, Norwich, UK; 3 Energy and Sustainability Research Institute Groningen, University of Groningen, Groningen, Netherlands; 4 Department of Earth System Sciences, Tsinghua University, Beijing, 100080 China; 5 State Environmental Protection Key Laboratory of Integrated Surface Water‐Groundwater Pollution Control, School of Environmental Science and Engineering, Southern University of Science and Technology, Shenzhen, China; 6 Guangdong Provincial Key Laboratory of Soil and Groundwater Pollution Control, School of Environmental Science and Engineering, Southern University of Science and Technology, Shenzhen, China; 7 Department of Mathematics, Southern University of Science and Technology, Shenzhen, China; 8 Centre for Climate and Environmental Policy, Chinese Academy for Environmental Planning, Beijing, China

**Keywords:** accounting, China, city, industrial ecology, inventory, water withdrawal

## Abstract

In the context of the freshwater crisis, accounting for water withdrawal could help planners better regulate water use in different sectors to combat water scarcity. However, the water withdrawal statistics in China are patchy, and the water data across all sectors at the city level appear to be relatively insufficient. Hence, we develop a general framework to, for the first time, estimate the water withdrawal of 58 economic–social–environmental sectors in cities in China. This methodology was applied because only inconsistent water statistics collected from different data sources at the city level are available. We applied it to 18 representative Chinese cities. Different from conventional perceptions that agriculture is usually the largest water user, industrial and household water withdrawal may also occupy the largest percentages in the water‐use structure of some cities. The discrepancy among annual household water use per capita in the urban areas of different cities is relatively small (as is the case for rural areas), but that between urban and rural areas is large. As a result, increased attention should be paid to controlling industrial and urban household water use in particular cities. China should specifically prepare annual water accounts at the city level and establish a timetable to tackle water scarcity, which is a basic step toward efficient and sustainable water crisis mitigation.

**Supplementary Information:**

The online version of this article (doi:10.1111/jiec.12999) contains supplementary material, which is available to authorized users.

## INTRODUCTION

Freshwater is a vital resource worldwide (Stewart, 2012; Showstack, 2013). The water resource per capita in China is only one‐quarter of the world average, and China is listed as one of the 13 water‐scarce nations around the world (Chapagain & Hoekstra, 2008; Liu et al., 2017). Meanwhile, rapid economic growth in China has led to large amounts of water use, and China has become the largest water user (Piao et al., 2010), compounding the adverse impacts of water pollution on water resource availability (Liu et al., 2017; Li et al., 2019a). As a result, two‐thirds of the cities in China suffer from freshwater scarcity (Qiao & Liu, 2014), and there are restrictions on the use of water by households and industries. It is predicted that the water‐use crisis in China will gain increasing attention (Zhao et al., 2012) due to reports that water demand in China will exceed the water supply by approximately 2030 (Shifflett et al., 2015).

Although China applied the most stringent water resource management system nationwide in 2011 to conserve water, including the Three‐Redline regulations in 2011 and the Water‐Ten in 2015, a draft was not proposed until June 2015, and only a few cities had begun installing water meters to record every drop since December 2017. Notably, if the volume of water withdrawal is implicit, it is difficult to regulate water demand, let alone eliminate the over extraction of water and assess the intensity of water use (such as the water consumption per industrial value added or the irrigation efficiency coefficient). The implicit volume of water withdrawal and water intensity creates more uncertainty and places constraint on sustainable economic development. Thus, it is important to account for water withdrawal to help planners better regulate water use in different sectors and fight against water scarcity. Herein, we prioritize the city level based on two considerations: (1) The city is the basic unit for water circulation between the economy and the environment and for evaluating water regulation policies (Li et al., 2019b). (2) Compared with the provincial and national dimensions, city‐level water use provides more disaggregated information. Therefore, collating and estimating sectoral water withdrawal data at the city level is a basic first step toward increasing water conservation. In this study, we propose a general methodology for establishing a water inventory for all economic–social–environmental sectors in prefectural cities in China. We disaggregate agriculture, industry, construction, services, household and environment into 58 subsectors.

Although the earliest water accounting studies appeared in the late 1950s, this field truly began to develop in the 2000s and has become somewhat popular in only the last decade, yet there are still few studies on this topic. Overall, the related research has evolved from including only a few sectors at its primary stage to the current accounts, which contain most economic–social–environmental sectors. Although Nace (1971) provided methods to record water use and establish commonly used accounting frameworks, it did not provide information on sectoral water use at any level. In recent decades, the California Federation (CALFED) program was developed in the Sacramento‐San Joaquin Delta of California to record water use (CALFED, 2018). This program originated in 1990 and developed over the next two decades. The CALFED program identified the concrete water demand of threatened fish species and is important because it generated a consensus on the need for timely and critical water withdrawal numbers that has become a regulatory baseline (CALFED, 2018). Based on this result, Brandt (2001) and Brown et al. (2009) continued to use this program to designate water demand for fisheries from 2001 to 2005 in USA.

Another important type of study, particularly focused on hydrological models, simulates sectoral water use worldwide (Flörke et al., 2013; Veldkamp et al., 2017; Wada et al., 2014); however, these are usually in a geographic grid unit rather than based on administrative territory. Moreover, they commonly regard construction, services, and households as a single sector called domestic water use (Alcamo et al., 2003), which omits water withdrawal information and difference of finer sectors in construction, services, and households.

Regarding the methodologies of specific‐sector studies, physical, hydrological, as well as economic methods have been developed. Baynes et al. (2010) used an integrated framework of stock and flow calculators in the water production sector and summarized the calibrations of historical water accounting systems. Okadera et al. (2015) focused on machining processes, including turning, milling, drilling, and cooling. Cazcarro et al. (2010) was based on a disaggregated social accounting matrix of Huesca in Spain. However, these investigations are limited to a few processes in a territory instead of all sectors in the economy. Thus, detailed water withdrawal in other sectors is rarely provided, indicating that these investigations are insufficient for exploring local water issues (Liu, Liu, & Yang, 2016). In addition, these water accounting calibrations also suffer from high variation, as they select different water sources (i.e., surface water, groundwater, and tap water) because the statistical water data in question were largely incomplete and only water withdrawal in part of a region or from a few kinds of water supply sources could be accessed. Finally, some industrial–ecology research applies life‐cycle‐based methods outside China, such as Owens (2001) and Berger and Finkbeiner (2013), but China’s water data are usually insufficient to apply the same method (Lin et al., 2012).

To date, sectoral water accounts have also been established in several countries at the national level, for example, Australia, Denmark, France, the Netherlands, New Zealand, Spain, and the United States (Maupin et al., 2014). Australia’s water account is one of the famous programs and presents water‐use information from 2000 to 2016 in Australia (Australian Bureau of Statistics, 2000–2016). However, this program still suffers from three problems: First, its data sources are disparate and originate from many different institutions, agencies and departments (Australian Bureau of Statistics, 2000–2016). Second, this program omits disaggregated information for construction, services, and environmental water use, as well as detailed industrial splits. Third, the data are incomplete and occasional due to the intermittent information used in early provisions (Baynes et al., 2010). In addition, AQUASTAT by the Food and Agriculture Organization (FAO) has also collected agricultural, industrial, and municipal water withdrawal by nation (FAO, 2019). However, they fail to disaggregate these water data into subsectors.

In sum, sectoral studies at the city level are still insufficient, and a sectoral water inventory of China’s cities has scarcely been attempted. There are several possible reasons for this gap. First, most water‐related statistics have concentrated on hydrology or water pollution, with few inventories focusing on water withdrawal. Second, some official statistics, such as those from the Chinese statistical bureaus, do not provide consistent or continuous statistics on water use no matter they are at the nation, province or city level. Third, the definitions of these statistics have frequently changed in terms of their calibration and period, reducing the possibility of comparison. Considering that these data cannot make up a systematic dataset, an accurate inventory of water withdrawal at the city level is still lacking that can fundamentally illustrate the water use of cities and improve regulations.

Thus, the contributions of this study are twofold. First, we develop a general methodology for constructing a water withdrawal inventory across the fifty‐eight sectors for cities in China. This methodology applies to many different circumstances for water statistics in different cities and provinces and covers the principal water supply sources (including surface water and groundwater). The framework is the first to combine incongruent water‐use data into one consolidated information set in a developing country, drawing on the China High Resolution Emission Gridded Dataset (Cai et al., 2018) and previous Water Resources Bulletins. Based on these sources, different sectors’ water withdrawals are made consistent and form an open water inventory, which allows us to evaluate the quality of the current data and identify data gaps for future improvement. Second, we applied this method to 18 representative cities in 2015 to analyze their water‐use characteristics. This may help planners obtain more accurate water statistics across the individual economic–social–environmental sectors driving water use, which can help governments better regulate water resources.

## ACCOUNTING FRAMEWORK FOR CITY‐LEVEL WATER WITHDRAWAL

### Scopes and boundaries for water accounts

**FIGURE 1:**
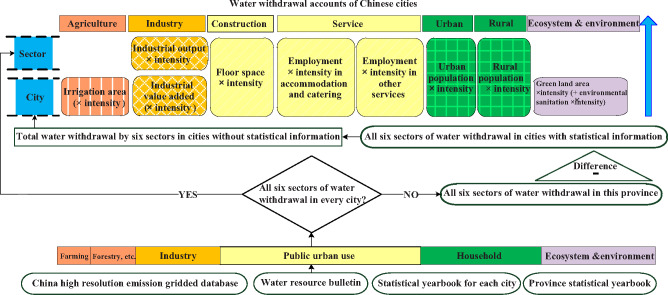
Water withdrawal inventory framework at the city level. *Note*. The circled sources at the bottom indicate the primary input for estimation.

The framework for the water inventory at the city level is illustrated in Figure 1 There are six sectors of water withdrawal illustrated by six different colors. Water withdrawal in this study is the water allocated to final users, including the water lost during delivery, and it mainly includes surface water and groundwater. According to the current statistical definitions of the Water Resources Bulletins at the city level, water withdrawal can be generally classified into six sectors as follows:

First, the *water used in farming* is the water consumed by paddy fields, irrigated croplands, and vegetable plots. Second, the *water used in forestry, animal husbandry, and fisheries* represents irrigation for forests and fruit trees, grassland preservation, fishpond maintenance, and cattle husbandry. For agricultural water withdrawal, we sum the two water withdrawal above. Third, for *industrial water withdrawal*, according to the China Statistical Yearbook, water withdrawal is the newly used water volume. This indicator may more accurately depict the dependence on the available domestic resources from economic activities because it excludes reused water. Industrial water withdrawal covers the demands of coal‐fired plants and nuclear power plants. Fourth, the *public water withdrawal in urban areas* is defined as the sum of the water withdrawal in construction and all service sectors. This definition of water withdrawal is a statistical feature that is different from other resource and economic statistics. Fifth, the *water withdrawal by household* consists of the water withdrawal by urban and rural residents in households. Finally, the *water withdrawal for ecosystem and environment preservation* includes grassland irrigation, deep well injection, environmental sanitation and improvements, and the supplementation of rivers, lakes, and marshes.

In addition, although each nation delivers its accounts differently, there is some similarity in terms of the structure and scope of water accounting, which is formalized in the handbook on the System of Environmental and Economic Accounting for Water Resources (UN, 2006). We basically comply with such defaults.

### Method

In Figure 1 each of the six sectors of water withdrawal is displayed in a unique color (and pattern) and organized from bottom to top vertically. There are three procedures as follows: (A) We looked for the water withdrawal for cities in a province by the six sectors from the provincial Water Resources Bulletin. We divided the provinces into two cases based on the available data.

Case 1. If the Water Resources Bulletin for this province provided water withdrawal for the six sectors for every administrative city, these data were compiled for later use (in the sectoral module). We allocated water withdrawal into each disaggregated subsector in Table S1‐1 in Supporting Information S1 for each city, including agriculture, industry, construction, services, urban households, rural households, and environmental water withdrawal.

Case 2. If the Water Resources Bulletin did not provide water withdrawal for the six sectors for all administrative cities, we then turned to the Water Resources Bulletin for each city to find water withdrawal for the six sectors for each city. For those cities that did not have these figures in their own Water Resources Bulletin, for each of the six sectors, we calculated the difference between the provincial magnitude and the sum of the water withdrawals for all cities that did have statistics in their city Water Resources Bulletins. Thus, we obtained the sum for each of the six sectors for all cities where water withdrawal for the six sectors was not included in their Water Resources Bulletin. The cities from Liaoning, Sichuan, Jiangsu, Zhejiang, Guangdong, Anhui, Hainan, Heilongjiang, Yunnan, and Jilin were categorized as Case 2 in 2015. The remaining cities were in Case 1.

(B) Case 2 leads us to the city module. Here, we allocated the sum of each sector for those cities without statistics; each sector had two multipliers, which are illustrated by the same color in Figure 1 that were selected as the driving forces of water use according to the current literature (Fan et al., 2019a). The logic behind this process is simple: We use a variable to multiply its water withdrawal intensity in the allocation. If the intensity was missing, we instead used these indicators to calculate the proportions to disaggregate the water withdrawal (see the detailed uncertainty analysis discussion below).

1. We used the irrigation water withdrawal per mu of farmland and the irrigation area to determine agricultural water use according to the data availability below.

Case 2.1. For cities with data for both the irrigation water withdrawal per mu (Intensity) and the irrigation area (Irriareas), we immediately obtain

1 $$ \mathrm{Wate}{\mathrm{r}}_{i,1}=\mathrm{Irriarea}{\mathrm{s}}_{i,1}\times \mathrm{Intensit}{\mathrm{y}}_{i,1} $$

As there is little uncertainty, this case is considered an advancement of previous studies such as Vardon et al. (2007), which used only the irrigation area by assuming that the irrigation water withdrawal per mu was equal among regions.

Case 2.2. If a city did not provide the irrigation water withdrawal per mu, we used the irrigation area instead, and we acknowledge that this does result in uncertainty (see detailed discussion below). In addition, because irrigation areas are close to the sown areas by definition, the sown areas could also be used in case that some cities did not provide the irrigation areas.

2 $$ \mathrm{Wate}{\mathrm{r}}_{i,1}=\mathrm{Irriarea}{\mathrm{s}}_{i,1}/\sum \limits_{i=1}^j\mathrm{Irriarea}{\mathrm{s}}_{i,1}\times \mathrm{Wate}{\mathrm{r}}_{j,1} $$

where *j* denotes the number of cities that did not provide figures in their own Water Resources Bulletins, and Water_*j*, 1_represents the sum of agricultural water withdrawal for those cities without statistical information.

2. Total industrial value added and water withdrawal per unit (Intensity) were used for industrial water withdrawal. Similar to Case 2.1, for cities with both indicators, we obtain

3 $$ \mathrm{Wate}{\mathrm{r}}_{i,k}=\mathrm{Valueadde}{{\mathrm{d}}_i}_{,k}\times \mathrm{Intensit}{\mathrm{y}}_{\mathrm{i},k},k\in \left[2,40\right] $$

In this case, there is also little uncertainty, which is a step beyond Guan et al. (2014), which assumed that industrial water withdrawal per value added was identical among regions. We regarded those cities with only value‐added data as Case 2.2.

3. We utilized the floor space of housing (Flospac) and the water withdrawal per unit (Intensity) to estimate water withdrawal for construction. For water withdrawal for accommodation and catering, which is usually the largest water user in the service sector, we assumed a positive correlation between water use and the number of employees and then used employment and water withdrawal per employee (Intensity).

4 $$ {\mathrm{Water}}_{i,41}^{\prime }=\mathrm{Flospa}{\mathrm{c}}_{i,41}\times \mathrm{Intensit}{\mathrm{y}}_{i,41} $$

5 $$ {\mathrm{Water}}_{i,44}^{\prime }=\mathrm{Employmen}{\mathrm{t}}_{i,44}\times \mathrm{Intensit}{\mathrm{y}}_{i,44} $$

6 $$ {\mathrm{Water}}_{i,k}^{\prime }=\mathrm{Employmen}{\mathrm{t}}_{i,k}\times \mathrm{Intensit}{\mathrm{y}}_{i,k},k\in \left[42,43\right]\cup \left[45,55\right] $$

4. We used the rural population (*Popul*, permanent residents) and household water withdrawal per capita in rural areas (*Intensity*) to estimate rural household water withdrawal. The estimation for urban household water withdrawal was quite similar, that is,

7 $$ {\mathrm{Water}}_{i,k}^{\prime }=\mathrm{Popu}{\mathrm{l}}_{i,k}\times \mathrm{Intensit}{\mathrm{y}}_{i,k} $$

8 $$ {\mathrm{W}}_{i,k}={\mathrm{W}\mathrm{ater}}_{i,k}^{\prime }/\sum \limits_{k=56}^{57}\mathrm{Wate}{\mathrm{r}}_{i,k},\mathrm{k}\in \left[56,57\right] $$

5. We used the area of green land, irrigation volume per green land area in urban areas (Intensity = 0.0782 m^3^), environmental sanitation areas (Sanitarea), and the water withdrawal per unit (Intensity′ = 0.0265 m^3^) to estimate ecosystem and environment water withdrawal, that is,

9 $$ \mathrm{Wate}{\mathrm{r}}_{i,58}=\mathrm{Greenlan}{\mathrm{d}}_{i,58}\times \mathrm{Intensit}{\mathrm{y}}_{i,58}+\mathrm{Sanitare}{\mathrm{a}}_{i,58}\times {\mathrm{Intensity}}_{i,58}^{\prime } $$

**C)** In the sectoral module, we utilized the disaggregated water withdrawal intensity and sectoral industrial output of each sector to divide the total industrial water withdrawal in each city (WaterIndus), that is,

10 $$ {\mathrm{W}}_{i,k}=\mathrm{Intensit}{\mathrm{y}}_{i,k}\times \mathrm{Outpu}{\mathrm{t}}_{i,k}/\sum \limits_{k=2}^{40}\left(\mathrm{Intensit}{\mathrm{y}}_{i,k}\times \mathrm{Outpu}{\mathrm{t}}_{i,k}\right) $$

11 $$ \mathrm{Wate}{\mathrm{r}}_{i,k}={\mathrm{W}}_{i,k}\times \mathrm{Wate}\mathrm{rIndu}{\mathrm{s}}_i,k\in \left[2,40\right] $$

Similarly, we used the proportions of water withdrawals (initial magnitude indicated by Water′) in construction, accommodation and catering and other services to separate urban and public water withdrawal. This procedure is more plausible than that used in Guan et al. (2014), which assumed that the water intensities of construction and all services were the same.

12 $$ \mathrm{Wate}{\mathrm{r}}_{i,k}={\mathrm{Water}}_{i,k}^{\prime }/\sum \limits_{k=41}^{55}{\mathrm{Water}}_{i,k}^{\prime}\times \left(\mathrm{Wate}{\mathrm{r}}_{i,\mathrm{UrbanPublic}}\right),k\in \left[41,55\right] $$

13 $$ \mathrm{Wate}{\mathrm{r}}_{i,k}={\mathrm{W}}_{i,k}\times \mathrm{Wate}\mathrm{rhousehol}{\mathrm{d}}_i,k\in \left[56,57\right] $$

### Data sources

The intensities of sectoral industrial water withdrawal were derived from the China High Resolution Emission Gridded Dataset (Cai et al., 2018), in which a key survey covers all the prefecture cities in China (294 by October 2017) and 39 industrial sectors (see Table S1‐1 in Supporting Information S1). Concretely, the intensities were calculated as the sectoral water withdrawals divided by the industrial output of sample enterprises (162,000 in total). The sectoral industrial outputs were taken from the Statistical Yearbook for each city. The irrigation water withdrawal per mu for farmland, industrial water withdrawal per value added, and household water withdrawal per capita in rural and urban areas were sourced from the Water Resources Bulletins at the province and city level.

The irrigation area, floor space of housing completed, employment, and green land area were sourced from the Statistical Yearbook of each province. Some other parameters, such as water withdrawal per floor space of housing completed (0.86 m^3^), representative water withdrawal intensity in accommodation and catering (718 liter/day/capita), intensity in other services (291.8 liter/day/capita), irrigation water withdrawal per green land in urban areas (0.0782 m^3^), and water withdrawal per environment and sanitation area (0.0265 m^3^), were sourced from the Bulletin of the First Water Resources Census (the Second Water Census of Shanghai) in 2011.

Therefore, a total of 22 variables were taken into account (Table Table 1) . For each city, changes in the intensity and variability of each parameter were considered. In concrete calibration, we tuned the water withdrawal according to local statistics, such as the gross water withdrawal in each of the sectors, which we believe gives a relatively accurate reflection of local water status. Even in Case 2.2, where not all data were available, we considered the discrepancies between cities and used intensities from an economically or demographically similar region to estimate cities at a similar stage because we believe that the local information is valuable and unique.

**TABLE 1:** The 22 variables and their source

	Agriculture	Irrigation areas○
		Irrigation water withdrawal per mu for farmland√
	Industry	Total industrial value added○/Δ
		Water withdrawal per industrial value added√
		Sectoral industrial outputΔ
		Disaggregated water withdrawal intensity of each sector※
	Construction	Floor space of housing○
		Water withdrawal per floor space of housingX
Services	Accommodation & catering	Number of employees in accommodation & catering∪
		Water withdrawal per employee in accommodation & cateringX
	Other services	Number of employees∪
		Water withdrawal per employee in other servicesX
Household	Rural	Rural population (permanent residents)○
		Household water withdrawal per capita in rural areas○/Δ
	Urban	Urban population (permanent residents)○
		Household water withdrawal per capita in urban areas○/Δ
	Environment & ecology	Green land areas∪
		Irrigation volume per green land area in urban areasX
		Environmental sanitation areasΔ
		Water withdrawal per unitX
		Livestock (for year 2013 and before)○
		Water withdrawal per cattle (for year 2013 and before)√

*Notes*. ^○^Province statistical yearbook.

^Δ^City statistical yearbook.

○/ΔProvince or city statistical yearbook or internet search.

^√^Water Resource Bulletins.

^※^China High Resolution Emission Gridded Database.

^X^Bulletin of the First Water Resources Census (the 2nd Water Census of Shanghai).

^∪^China City Statistical Yearbook.

There are 58 industries in total. As illustrated by the shaded sectors in Supporting Information S1, 2–7 are mining and processing, 8–37 are manufacturing, 38–40 are production and supply of electricity, gas and hot water and 42–55 are services. These were selected based on the National Accounting System and are widely used as industrial classifications.

### Uncertainty analyses

We estimated the sensitivity of water withdrawal by agriculture, industry, and services for cities in Case 2.2 by replacing the specific intensity value with regional, provincial, or national magnitudes based on data availability. We take the cities of Anhui, Jiangsu, and Zhejiang as examples because their Water Resources Bulletin did not provide water withdrawal for the six sectors for some of their cities.

For agricultural water withdrawal in Case 2.2, one assumption was applied for cities without statistical information: If there was no available irrigation water withdrawal per mu for farmland, the water withdrawal intensity for the agriculture sector would be the same. We conducted a sensitivity analysis by replacing the intensity with the regional or provincial values, the results of which are shown below:

Although the largest differences between *Agricultural water withdrawal’* (estimated with the replaced agricultural water withdrawal intensity) and our original estimation appeared in Wuxi and Changzhou (−14.3%) in Table 2 the average variation of the absolute value was 9.0%. This result indicates that there are no substantial differences, and the method in Case 2.2 provides a credible estimation of agricultural water withdrawal.

**TABLE 2:** Sensitivity analysis of agricultural water withdrawal

City	Agricultural water withdrawal (10 thousand m^3^)	Irrigation area (1,000 hectare)	Irrigation water withdrawal (m^3^)/mu for farmland	Agricultural water withdrawal (10 thousand m^3^)	Uncertainty
Hefei	150,301	458	282	129,452	0.139
Bengbu	77,757	237	282	66,971	0.139
Huaibei	46,844	143	282	40,346	0.139
Tongling	26,785	82	282	23069	0.139
Huangshan	16,797	51	282	14,467	0.139
Suzhou	138,363	422	282	119,170	0.139
Xuancheng	65,793	201	282	56,666	0.139
Wuxi	60,956	173	461	71,123	−0.143
Xuzhou	408,634	1,161	389	402,364	0.016
Changzhou	75,578	215	461	88,184	−0.143
Nantong	294,246	836	389	289,806	0.015
Lianyungang	223,122	634	389	219,698	0.016
Huai’an	280,145	796	389	275,847	0.016
Zhenjiang	83,091	236	389	81,838	0.015
Taizhou	204,571	581	389	201,484	0.015

For the industrial water withdrawal estimation, although the largest differences between *Industrial water withdrawal’* (estimated with the replaced water withdrawal intensity) and our original estimation appeared in Xuzhou, Lianyungang, and Huai’an (−13.5%) in Table 3 the average variation in the absolute value was 7.3%. This result indicates that there is relatively low sensitivity, and the method in Case 2.2 provides a credible estimation of industrial water withdrawal. Moreover, the cities were the same as those selected for the validation of agricultural water withdrawal, which also supports the robustness of our method.

**TABLE 3:** Sensitivity analysis of industrial water withdrawal

City	Industrial water withdrawal (10 thousand m^3^)	Industrial value added (100 million yuan)	Industrial water withdrawal (m^3^)/10000‐yuan value added	Industrial water withdrawal (10 thousand m^3^)	Uncertainty
Hefei	96,390	2,256	45	101,053	−0.048
Bengbu	29,050	680	45	30,455	−0.048
Huaibei	21,425	501	45	22,462	−0.048
Tongling	18,478	432	45	19,372	−0.048
Huangshan	5,425	127	45	5,687	−0.048
Suzhou	15,747	369	45	16,509	−0.048
Xuancheng	17,585	412	45	18,436	−0.048
Wuxi	52,013	2,953	15	50,176	0.037
Xuzhou	47,705	2,709	18	55,163	−0.135
Changzhou	46,050	2,615	15	44,423	0.037
Nantong	51,115	2,902	14	46,697	0.095
Lianyungang	20,368	1,157	18	23,552	−0.135
Huai’an	26,122	1,483	18	30,206	−0.135
Zhenjiang	35,767	2,031	14	32,676	0.095
Taizhou	43,419	2,465	14	39,667	0.095

*Note*. For cities in Anhui, we could obtain only the industrial value added for the above‐designated‐sized enterprises.

Similarly, for services, we assumed that the water withdrawal per employee would be equal within a city (for cities with statistical information) or among cities (for those without). We estimated the uncertainty of other services using water withdrawal per employee at the national level from the cities with statistical information. The list of cities used is provided in Appendix II. We observed that the average variation was 8.0%. This result indicates that there are no large differences, and the method in Case 2.2 provides an accurate and credible method for estimating service water withdrawal. In fact, similar proportions were also used in the estimations of the China Institute of Water Resources and Hydropower Research (Gan et al., 2013; Xie et al., 2015; Zhang et al., 2013). The validation process for households and the environment are similar, and we omit this part here and make it available upon request.

Finally, given that China did not officially report statistics on environmental sanitation areas at this stage, we omitted the validation of environmental water withdrawal. Nevertheless, this will be possible when such data are available, considering that many cities are beginning to explore how to estimate their environmental sanitation areas.

Estimating city‐level water withdrawal by deducing or scaling down the numbers from the administrating‐province statistics was not feasible because the sectoral water withdrawal data availability at the province level was even worse than that at the city level. For example, for 2007, many studies such as Guan et al. (2014) and Zhao et al. (2015) were still using the 2008 data from the Chinese Economic Census Yearbook as a substitute for 2007 data due to a lack of figures, and these results could suffer from bias as there may have been a structural change in resource use before and after 2008 (Yuan et al., 2010). Even worse, water withdrawal information was no longer included in the Chinese Economic Census Yearbook 2013, which was the edition following 2008. Similarly, another indicator of agricultural water withdrawal, the cultivated land area, was considered, but we excluded this indicator given that it did not count the number of planting seasons and thus was unable to accurately reflect water withdrawal information.

## RESULTS AND DISCUSSIONS

### Applications of the methodology

**FIGURE 2:**
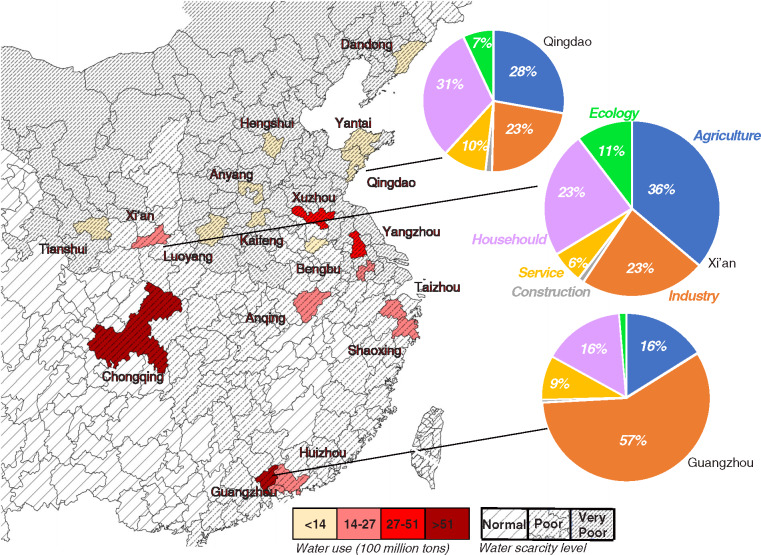
Water withdrawals in the 18 cities in 2015. Underlying data used to create this figure can be found in Supporting Information S2

We applied the methodology to 18 cities in 2015, as listed in Table 4. These cities represent 11 provinces, 6 economic zones, and 5 regions of China and contain some metropolitan areas (such as the provincial capitals Guangzhou and Chongqing), coastal cities (such as Qingdao), and undeveloped cities (such as Kaifeng) around different basins. Furthermore, we related these cities to the water scarcity assessment according to the method used in Liu et al. (2017). Figure 2 depicts the geographic distribution of these 18 cities and the typical water‐use structure of three of them. For simplicity, in the map, *Normal* water scarcity levels (the dense lines) indicate that there is neither quantity‐ nor quality‐induced scarcity in these cities, *Poor* (the denser lines) indicates there is only quality‐induced water scarcity, while *Very Poor* (the densest lines) indicates both quantity‐ and quality‐induced water scarcity. For more information, refer to the method developed by Liu et al. (2017) based on Zeng et al. (2013). In total, there were seven cities in the *Poor* classification, ten cities were categorized as *Very Poor*, and one was categorized as *Normal*. Several characteristics of water withdrawal could be drawn from the perspectives of both city and industry.

**TABLE 4:** Water withdrawal and socioeconomic index of 18 cities in 2015

City	Province	Representative economic zone	Region	Water scarcity assessment	Ww (10 k m^3^)	Water intensity (m^3^/10 k yuan)	GDP/capita (yuan)	Population (10 k)	Ww/capita (m^3^)	Household Ww/capita (m^3^)
Hengshui	Hebei	Jing‐Jin‐Ji	North	Very poor	30,095	25	27,543	452	67	6
Yantai	Shandong	Central Bohai Bay			86,900	13	91,979	653	133	21
Qingdao					87,572	9	102,519	782	112	35
Dandong	Liaoning				97,949	99	40,850	239	410	38
Tianshui	Gansu	Western zone	West		37,058	66	16,956	331	112	17
Chongqing*	Chongqing			Normal	951,360	61	52,321	3,374	282	40
Xi’an*	Shaanxi			Poor	182,036	31	66,938	815	223	52
Huizhou	Guangdong	Pearl River Delta	South		205,924	66	66,231	353	584	97
Guangzhou*					661,400	37	136,188	848	780	122
Shaoxing	Zhejiang	Yangtze River Delta	East		196,219	44	90,003	443	443	57
Taizhou					188,589	53	58,917	597	316	45
Xuzhou	Jiangsu			Very poor	511,474	96	61,511	1,026	498	17
Yangzhou					396,227	99	89,647	461	859	60
Kaifeng	Henan	Central zone	Center		131,580	82	35,326	551	239	21
Luoyang					141,300	41	51,692	698	202	30
Anyang					143,550	76	36,828	614	234	32
Anqing	Anhui			Poor	270,200	191	31,101	622	434	35
Bengbu					124,235	99	38,267	374	332	34
Total	11	6	5	3	18	18	18	18	18	18

*Note*. Ww is short for water withdrawal.

^*^indicates a capital‐level city.

Table 4 shows the water withdrawal index and other socioeconomic characteristics of the 18 cities. Some cities, such as Hengshui, Yantai, and Qingdao, have less water withdrawal per capita and lower water withdrawal intensity than others. Apart from a less water‐consumptive production structure (Li et al., 2019b), some other possible reasons for this difference could be that these areas have advanced water conservation technology. For example, Hengshui is aimed to become a pilot city for water conservation in December 2018, and it has broadly applied drip and spray irrigation in modern agriculture parks. There are two above‐100,000‐ metric‐ton desalination factories in Qingdao, which represent cutting‐edge technology for China. In addition, mulch planting and the integration of water into fertilizer have also been developed in the cities of Yantai and Qingdao city. For more information, see in References websites of China Daily and the people’s government of Hebei province.

### Industrial and household water use may also occupy the largest percentages

**FIGURE 3:**
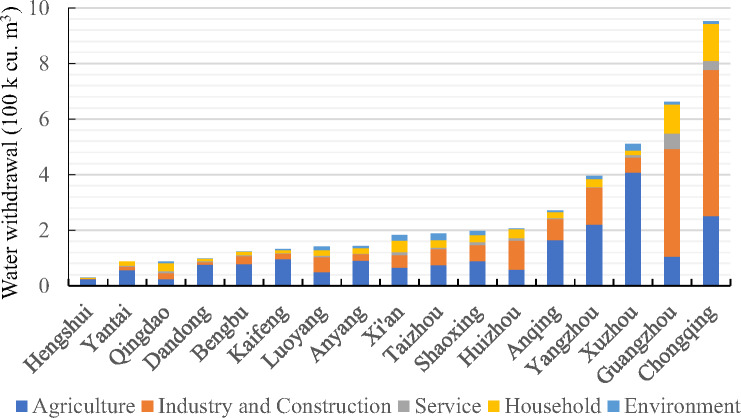
Water withdrawal by city and sector in 2015. Underlying data used to create this figure can be found in Supporting Information S2

In Xi’an, Shaoxing, Taizhou, Luoyang, and Chongqing, the shares of agricultural water withdrawal are less than 50%, indicating that industry and household water withdrawals in these areas are beginning to dominate the water‐use structure. First, industrial water withdrawal also occupies a large percentage in the water‐use structure of some cities, although the average share of agricultural irrigation is 58.5% across the 18 cities. Contrasting Hengshui and Luoyang, the water withdrawal of agriculture in Hengshui is highest (86%) and lowest in Luoyang (31.4%); conversely, the share of industrial water withdrawal is high in Luoyang (49.7%) and low in Hengshui (7.8%). Second, in Guangzhou and Qingdao, household water withdrawal is greater than agricultural water withdrawal, as shown in Figure 3 This point is also supported by evidence from the China City Statistical Yearbook 2013, as urban household water use accounted for 36% of the total water use for all prefecture cities in China.

### A comparison of top water‐use sectors at the city level after agriculture

**FIGURE 4:**
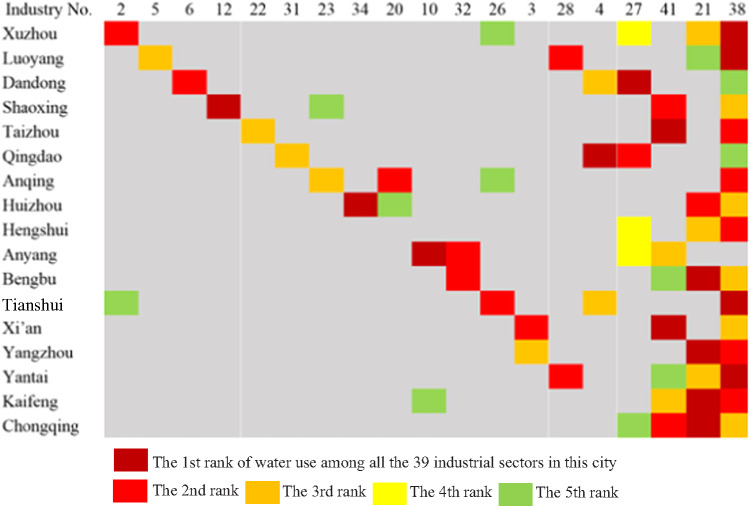
Similarities between the top water‐use industries. Underlying data used to create this figure can be found in Supporting Information S2

The principal water‐use sectors are similar for different cities. Overall, manufacturing and electricity, gas and hot water take the first two places after agriculture, as shown in Figure S1‐1 in Supporting Information S1, although the water in hydropower is also reused by the downstream. We ranked the water withdrawals of the secondary sectors in each city and identified the top three water users for each city, as indicated by the numbers and squares in Figure 4 The production and supply of electricity and hot water (No. 38) is ranked among the top three industrial water uses in 17 of the 18 cities, and raw chemical materials and products (No. 21) is among the top three in eight cities. The most red and yellow squares are located at the upper‐right side of Figure 4 indicating the similarity in the water‐use industries.

For the industrial sectors, the high users at the city level include smelting and pressing of ferrous metals and mining and processing of ferrous metal ore, after the production and supply of electricity and hot water and raw chemical materials and products. Water is most used in industrial processes, such as mining, processing, cooling, air conditioning, clarifying, and washing (Fan et al., 2019b, 2019c; Li et al., 2019b). Similarly, the three services with the highest water use at the city level are accommodation and catering, education, and public management, social security. The main reason for this finding may be that there is more water‐use infrastructure for public services or leisure activities in these sectors (Gössling, 2001), such as bathing, swimming, car washing, piped channels, and other carriers in schools.

### The significant discrepancy between urban and rural household water withdrawal

The total annual household water withdrawal per capita (AHWUPC) varies from 6 m^3^ in Hengshui to 122 m^3^ in Guangzhou; this variation could be attributed to the significant difference in AHWUPC between urban and rural areas, caused by the relatively high urbanization rate and improved living standards in cities such as Guangzhou. First, we observe that the discrepancy in the AHWUPC in urban areas of different cities is small, especially within a province, due to shared development policies. The AHWUPC in urban areas ranges from 33 m^3^/capita in Anyang to 55 m^3^/capita in Anqing, indicating that the difference in urban AHWUPC from one city to another is not as significant as could have been imagined, which is also the case for the difference in the rural AHWUPC. However, the discrepancy between urban and rural areas is relatively large: the urban AHWUPC is 1.36 times the rural on average and 11 times the maximum in both Bozhou and Lu’an city. From this perspective, it would be meaningful to explore different lifestyles and water withdrawal per capita.

## CONCLUSIONS AND IMPLICATIONS

In this study, a methodology was developed to estimate the water withdrawal of 58 economic–social–environmental sectors for cities in China based on the China High Resolution Emission Gridded Database and previous Water Resources Bulletins. This methodology can be applied to the different water statistics collected from cities and provinces; here, six consistent water‐use sectors are used, which helped in combining separate water‐use data into one consolidated information set. Based on the inventory, we identified some characteristics of water withdrawal from the perspectives of both the city and the sector, which aimed at addressing concerns about the current and future state of water resources in China and helping to combat the water crisis. Some policy implications are as follows.

First, the control of industrial and urban household water use deserves increased attention. Considering that industrial water withdrawal dominates the water‐use structure in some cities, China should manage water during industrialization efficiently and sustainably. In addition, more attention should be paid to cities with higher urbanization rates or better living standards because these factors could make a large difference in household water withdrawal in a city. Thus, controlling urban household water withdrawal may become increasingly important for reducing household water use.

Second, China should improve its water statistics and specifically prepare annual water accounts at the city level. Water accounts can be applied to investigate the impacts of changes in water resource allocation and use, including assessment of the influence of structural changes and economic development (such as Cole, 2004; Jia et al., 2004), the driving forces from different industries behind particular water problems (Rijsberman, 2006; Vörösmarty et al., 2000), and the sectoral impacts of water regulation (including charges and incentives) (Jønch‐Clausen & Fugl 2001; Saleth & Dinar, 2000). Historically, due to a lack of disaggregated water data at the sector or city level, this type of research was insufficient, but it now has the potential to be developed. In addition, water inventory data could help domestic water users better comply with the Three‐Redline regulations because it is difficult to reach a target without comparable water use numbers. Data would also increase transparency because in China, some officials with responsibility for water use may feel pressure to reveal water‐use data to the public because these data are included in the performance evaluation system for political promotion, and they care about their own achievement. For details, see Beech (2015).

Third, the most water‐use industries should be targeted at the city level to improve water management. Information from the water inventory provides a window through which different cities can learn from one another in terms of promoting water conservation technology.

Although we searched for the most solid estimation methods based on the available bulletins and statistics, there are still some limitations to this study due to the defects in the sectoral figures.

First, we did not split out irrigation for forests, fruit trees, grassland preservation, fishpond maintenance, or cattle husbandry from water withdrawal in the forestry, animal husbandry, and fisheries sectors or for different crops. There is thus some potential to improve the water‐use inventory when more disaggregated parameters are accessible, such as water withdrawal per floor space of housing completed, irrigation water withdrawal per area of green land in urban areas, water withdrawal per environment and sanitation area, and representative water withdrawal for accommodation and catering and for other services. In sum, this methodology does not solve all problems; instead, it delivers an essential tool for addressing these issues.

Second, water withdrawal may be affected by variations in domestic precipitation (Yureklwe & Kurunc, 2006) and occasional hydrological disasters. For example, it is common for one city to use more water to combat drought, especially for the water used for agriculture and that for ecosystem and environment preservation. This is different from other resource data such as energy consumption. This is an unneglectable characteristic of water accounting. Thus, sectoral water withdrawal may change by a large proportion even in adjacent or economically similar cities. These variations create high uncertainty in the estimations of the cities in question. As precipitation usually displays a high level of spatial and temporal variability (Yu et al., 2007; Zhou & Yu, 2005), it would be meaningful to study the effects of precipitation on water use due to the urban rain island effects (Yu et al., 2017). Finally, in the future, we would like to work on time series data set to further check the robustness of this methodology with data for 2008, 2010, 2012, and 2015, respectively.

## Supplementary Information

**Supporting Information S1**: This supporting information S1 includes a table with a list of the 58 sectors in this study, a list of 20 cities used to calculate the water withdraw per service employee from the cities with statistical information at the national level, a figure with the structure of industry and service water use and the top water‐use sectors.

**Supporting Information S2**: This supporting information S2 provides data plotted in Figures 2 4 of the main text.
