# Supplementary material for: City‐level water withdrawal in China: Accounting methodology and applications
Source: J Ind Ecol. 2020 Mar 9;24(5):951–64. doi: 10.1111/jiec.12999 (PMC13068713; doi:10.1111/jiec.12999)
Supplement: Supplementary file 1 — Supporting Information S1: This supporting information S1 includes a table with a list of the 58 sectors in this study, a list of 20 cities used to calculate the water withdraw per service employee from the cities with statistical information at the national level, a figure with the structure of industry and service water use and the top water‐use sectors. [file 44498_2020_2405003_MOESM1_ESM.pdf]

## SUPPORTING INFORMATION FOR:

Zhang, Z., J. Liu, B. Cai, Y. Shan, H. Zheng, X. Li, X. Li, and D. Guan.
2020. City-level Water Withdrawal in China: Accounting Methodology and Applications. *Journal of Industrial Ecology.*

**Summary**

This supporting information S1 includes a table with a list of the 58 sectors in this study, a list of 20 cities used to calculate the water withdraw per service employee from the cities with statistical information at the national level, a figure with the structure of industry and service water use and the top water-use sectors.

## I. **Table S1-1:** A list of the 58 sectors in this study

| | | | |
|---|---|---|---|
| 1 | Farming, Forestry, Animal Husbandry, and Fishery | 30 | General Purpose Machinery |
| 2 | Coal | 31 | Special Purpose Machinery |
| 3 | Extraction of Petroleum and Natural Gas | 32 | Transport Equipment |
| 4 | Ferrous Metal Ores | 33 | Electrical Machinery and Equipment |
| 5 | Nonferrous Metal Ores | 34 | Communication Equipment, Computers, and Other Electronic Equipment |
| 6 | Nonmetal Ores | 35 | Measuring Instruments and Machinery for Cultural Activity and Office Work |
| 7 | Other Minerals | 36 | Other Manufacture |
| 8 | Processing of Food from Agricultural Products | 37 | Comprehensive Utilization of Waste Resources |
| 9 | Foods | 38 | Electricity and Hot Water |
| 10 | Liquor, Beverage, and Refined Tea | 39 | Gas |
| 11 | Tobacco | 40 | Tap Water |
| 12 | Textile | 41 | Construction |
| 13 | Textile Wearing Apparel and Caps | 42 | Wholesale, Retail Trade |
| 14 | Leather, Fur, Feather, and Related Products and footwear | 43 | Transportation, warehousing, and postal industry |
| 15 | Processing of Timber, Wood, Bamboo, Rattan, Palm, and Straw Products | 44 | Accommodation and Catering Industry |
| 16 | Furniture | 45 | Information transfer, Computer Service, and Software Industry |
| 17 | Paper and Paper Products | 46 | Financial industry |

| | | | |
|---|---|---|---|
| 18 | Printing, Reproduction of Recording Media | 47 | Real estate |
| 19 | Culture, Education, Handicraft, Fine Arts, Sports, and Entertainment Articles | 48 | Leasing and Business Services |
| 20 | Processing of Petroleum, Coking, and Nuclear Fuel | 49 | Scientific research and technical services |
| 21 | Raw Chemical Materials and Products | 50 | Water, environment, and public facilities management |
| 22 | Medicines | 51 | Resident services and other services |
| 23 | Chemical Fibers | 52 | Education |
| 24 | Rubber | 53 | Health and social work |
| 25 | Plastics | 54 | Culture, sports, and entertainment |
| 26 | Nonmetallic Mineral Products | 55 | Public Management, Social Security, and Social welfare |
| 27 | Smelting and Pressing of Ferrous Metals | 56 | Urban |
| 28 | Smelting and Pressing of Nonferrous Metals | 57 | Rural |
| 29 | Metal Products | 58 | Environment and ecology |

**II.** A list of 20 cities used to calculate the water withdraw per service employee from the cities with statistical information at the national level:

Xiamen City;

Shenzhen City;

Zhengzhou City;

Qingdao City, Laiwu City;

Lianyungang City, Huai'an City;

Wuhan City, Huangshi City, Shiyan City, Yichang City, Xiangyang City, Ezhou City, Jingmen City, Xiaogan City, Jingzhou City, Huanggang City, Xianning City, Suizhou City, and Qianjiang City.

**III.**

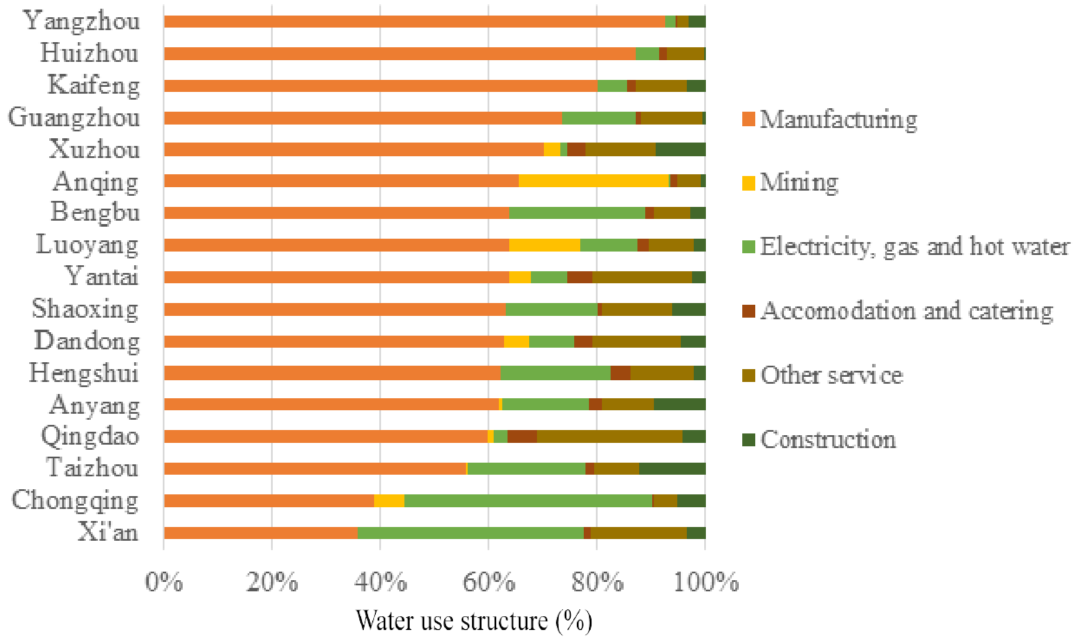


**Figure S1-1:** The structure of industry and service water use and the top water-use sectors.
